# Bedrock-Dependent Effects of Climate Change on Terricolous Lichens Along Elevational Gradients in the Alps

**DOI:** 10.3390/jof10120836

**Published:** 2024-12-03

**Authors:** Chiara Vallese, Luca Di Nuzzo, Luana Francesconi, Paolo Giordani, Daniel Spitale, Renato Benesperi, Gabriele Gheza, Petra Mair, Juri Nascimbene

**Affiliations:** 1 Department of Earth, Environment and Life Sciences (DISTAV), University of Genova, Corso Europa 26, 16132 Genova, Italy; chiara.vallese@unige.it; 2 BIOME Lab, Department of Biological, Geological and Environmental Sciences, Alma Mater Studiorum University of Bologna, Via Irnerio 42, 40126 Bologna, Italy; luana.francesconi3@unibo.it (L.F.); gabriele.gheza@unibo.it (G.G.); juri.nascimbene@unibo.it (J.N.); 3 Department of Pharmacy, University of Genova, Viale Cembrano 4, 16148 Genova, Italy; giordani@difar.unige.it; 4 Museo di Scienze Naturali Dell’Alto Adige, Via Bottai, 1, 39100 Bolzano, Italy; spitale.daniel@gmail.com (D.S.); petra.mair@naturmuseum.it (P.M.); 5 Dipartimento di Biologia, University of Florence, Via la Pira 4, 50121 Florence, Italy; renato.benesperi@unifi.it

**Keywords:** beta-diversity, climate change, cryophylous species, Dolomites, functional diversity, Rhaetian Alps, species richness, terricolous lichens

## Abstract

In this study, we focused on the bedrock-dependent effects of climate change on terricolous lichen communities along elevational gradients in the Alps. In particular, we contrasted between carbonatic and siliceous bedrock, hypothesizing more favourable conditions on siliceous than on carbonatic bedrock, where dryer conditions may exacerbate the effects of climate change. To test this hypothesis, we compared terricolous lichen diversity patterns between the two bedrock types in terms of (1) species richness, (2) beta-diversity, (3) proportion of cryophilous species, and (4) functional diversity, also testing the effect of the elevational gradient as a proxy for expected climate warming. Our results indicate that the most cold-adapted part of the terricolus lichen biota of the Alps could be especially threatened in the near future, mainly on carbonatic bedrock. Actually, contrasting diversity patterns were found between carbonatic and siliceous bedrock, clearly revealing a bedrock-dependent effect of climate change on terricolous lichens of the Alps. As hypothesized, siliceous bedrock hosts a richer lichen biota than carbonatic bedrock, reflecting a general richness pattern at the national level. In general, siliceous bedrock seems to be less prone to rapid pauperization of its lichen biota, providing more suitable climatic refugia that can mitigate the effects of climate warming on terricolous lichens.

## 1. Introduction

Human-induced climate change is one of the main drivers of species loss worldwide, and has been estimated to cause the extinction of macroscopic species in the range of 14–32% before 2100, even in intermediate greenhouse gas emission scenarios [1]. Depending on numerous factors, the effects of climate change on biodiversity and ecosystems vary significantly [2], with certain biomes being more negatively affected than others, such as the alpine ecosystems, where limited space is available for the upward shift of species to track climate change. Additionally, alpine species often occupy specific climatic niches, making them highly susceptible to even minor environmental changes, resulting in considerable negative impacts [3,4]. This has led to the evolution of highly specialised organisms adapted to harsh climatic conditions [4,5,6]. The high rate of specialised species, combined with the faster warming at higher elevations, pose a disproportionate threat to biodiversity in alpine ecosystems [4,7,8]. In the European Alps, air temperatures are expected to rise, accompanied by a significant reduction in the duration of snow cover and more complex variations in precipitation patterns [9].

Exhaustive predictions of biodiversity responses to climate change should incorporate local factors that may interact with climate change [10,11], as in the case of bedrock type in alpine ecosystems [12], where the physical structure of the bedrock influences plant species composition due to its effect on water percolation and soil moisture availability [10,12,13,14]. Underlying rocks can affect the chemical composition of the soil, its pH, and even its structure. Different siliceous and carbonatic soils have different underlying rock, with different physics and chemical characteristics. This applies to porosity and fracturing, which affect the maintenance of moisture on the surface soil layer. In general, the higher water availability on siliceous bedrock creates a more mesic environment due to higher water and nutrient supply at the subsurface level, which fosters highly competitive but poorly stress-tolerant species [14,15,16]. In contrast, carbonatic bedrock tend to be drier, likely due to the high porosity of the rock, which enhances rapid runoff. The higher erosion potential of carbonatic bedrocks as compared to siliceous ones, coupled with limited water availability, drives the selection of more stress-tolerant and less competitive species [10,12].

While bedrock-dependant effects of climate change are relatively well elucidated for vascular plants (e.g., [10,12,16,17]), information on other sessile organisms with different ecophysiology is still scarce, as in the case of terricolous lichens. In general, lichens are poikilohydric organisms that are not able to actively control their water content [18]. Environmental moisture conditions play a pivotal role in regulating thallus saturation and desiccation cycles, thus influencing metabolic processes. From this perspective, climatic factors, including air temperature and precipitation, strongly influence respiration and photosynthesis [18,19], determining high susceptibility of lichens to climate change [20,21]. Such susceptibility might also arise from altered biotic conditions. In mountain ecosystems, the upward shift of more thermophilous and competitive vascular plants could determine a reduction in lichen diversity due to increasing competition [22].

At the same time, the effects of abiotic and biotic conditions on lichen diversity are mediated by their functional traits [23,24]. For example, the combination of different thallus structures (growth forms), photobiont identity (cyanobacteria or green algae), and the presence of carbon-concentrating mechanisms enables lichens to thrive across diverse environments with different water sources and temperature and light conditions [25,26]. Thus, considering functional traits allows us not only to better comprehend the responses of the organisms to changes in environmental conditions, but also to link community changes to ecosystem functioning [24].

While lichens are known to rely on atmospheric moisture, terricolous species are likely affected also by soil water retention capacity, especially crustose species that are partially embedded into the soil or squamulose and foliose species that are attached through rhizines [27,28]. Besides their strong relationship with environmental and bedrock conditions, in alpine ranges, terricolous lichens are among the main components of biological soil crusts that greatly contribute to ecosystem biodiversity and functioning [29,30,31,32] and trigger colonization processes on bare soils, as is the case with recently deglaciated areas [33]. Despite their relevant ecological role, the response of these lichens to climate change in terms of diversity patterns that can be mediated by the bedrock type (e.g., carbonatic vs. siliceous bedrock) is still scarcely explored.

In this study, we focused on the bedrock-dependent effects of climate change on terricolous lichens along elevational gradients in the Alps. Elevational gradients are a valuable tool for studying the impacts of climate change on biodiversity, as various climatic factors vary over short distances, mimicking the variations expected under climate change scenarios [34,35]. In particular, we contrasted between carbonatic and siliceous bedrock, hypothesizing more favourable conditions on siliceous than on carbonatic bedrock, where dryer conditions may exacerbate the effects of climate change. To test this hypothesis, we compared terricolous lichen diversity patterns between the two bedrock types in terms of (1) species richness, (2) beta-diversity, and (3) proportion of cryophilous species, and (4) functional diversity, also testing the effect of the elevational gradient as a proxy for expected climate warming.

## 2. Materials and Methods

### 2.1. Study Area

The study was carried out in the Central-Eastern Italian Alps, from the Rhaetian region to the Dolomites [36]. The survey on carbonatic bedrock was carried out in the Dolomites (Southeastern Alps), including the provinces of Bolzano (BZ, South Tyrol), Trento (TN, Trentino), and Belluno (BL, Veneto). The Dolomites are a group of mostly Triassic carbonatic mountains with several peaks over 3000 m elevation. The mean annual precipitation is 1050 mm, while at an elevation of 2000 m, the mean minimum temperature ranges between −8 °C (January) and +7 °C (July), and the mean maximum temperature ranges between −2 °C (February) and +15 °C (July–August) [37]. The survey on siliceous bedrock was carried out in the Eastern and Western Rhaetian Alps, in the province of Bolzano. The area is characterized by complex geology, including magmatic, sedimentary, and metamorphic units. The latter belong to both basement and marine stratigraphic succession, dating from the Permian up to the Cenozoic. The average annual temperature recorded at the Careser meteorological station (2607 m) during the 1961–1990 period is −1.2 °C. February is the coldest month with a temperature of −8.3 °C, while July is the warmest at +6.9 °C. The mean annual precipitation is 928 mm, with the minimum in winter and the maximum in late spring–summer. Winter (December–February) is the driest season (140 mm), while summer (June–August) is the wettest (288 mm) [38].

### 2.2. Sampling Design, Specimen Collection, and Identification

**Figure 1:**
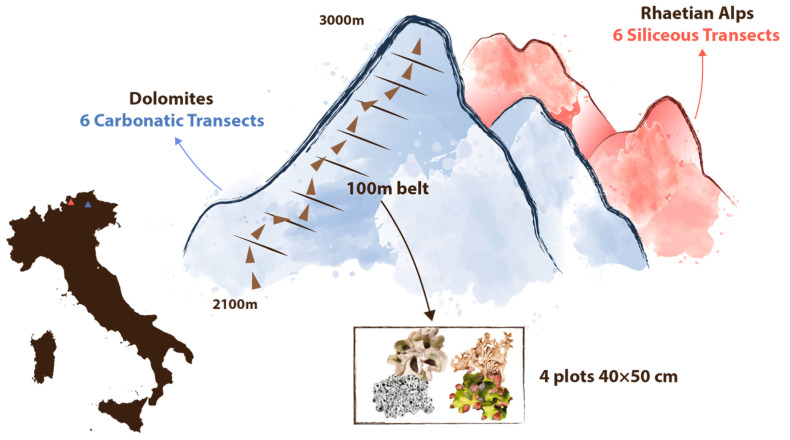
Location of the study area with a conceptualization of the sampling design. We selected 6 transects on carbonatic and 6 on siliceous bedrock. Each elevational transect was split into 100 m belts. In each belt, lichens were recorded in 4 randomly selected rectangular (40 × 50 cm) plots. This figure was assembled using Adobe Illustrator 2024.

The field survey was carried out in summer 2018 and 2019. The sampling design, originally conceived for the BRIOCOLL Project, included 394 plots and 98 elevational belts, surveyed in 12 independent elevational transects ranging from 2100 to 3000 m of altitude. Six transects were placed on carbonatic bedrock (Dolomites) along the slopes of Sasso Lungo (SL), Pisciadù (PI), Cunturines (CO), Lagazuoi (LA), Croda del Becco (CB) and the saddle of Antersass (AN). Six transects were established on siliceous bedrock (Eastern and Western Rhaetian Alps), on the eastern and western sides of Val Mazia (PE, PW), Val di Roia (RE, RW), and Val Martello (ME, MW). Each transect was split into 100 m elevational belts, and lichen occurrences were recorded in 4 plots (40 × 50 cm) in each belt (Figure 1). In some transects, it was not possible to sample the entire elevational gradient (2100 to 3000 m), since, in some cases, the lower elevation belts were covered by forests, and in others, the maximum accessible elevation was below 3000 m. Vascular vegetation cover was similar on the two substrata and decreased linearly with altitude at the same rate. Since the Dolomites are lithologically heterogeneous and complex, our sampling transects were strictly allocated only in areas with calcareous or dolomitic bedrock, thus avoiding areas with porphyritic, volcanic basic, or terrigenous rocks. In the Rhaetian Alps, we allocated our sampling transect only to areas with either magmatic or siliceous metamorphic rocks (gneiss, schists with quartz).

Since correct identification in the field was almost impossible for most of the species, we systematically collected specimens for laboratory identification. Specimens were analysed using dissecting and a standard light microscope. Routine chemical spot tests were also performed for most specimens. The identification of sterile crustose lichens (including all Lepraria species) was based on standardized thin-layer chromatography (TLC) analyses with solvents A, B’, and C, following the protocols of [39]. Nomenclature followed [40]. The specimens were stored in the herbarium BOLO (lichen collection by J. Nascimbene).

### 2.3. Statistical Analyses

Occurrence data were aggregated at the belt level, resulting in a presence/absence data frame of species for each elevational belt. For alpha diversity, we used the species richness, which was calculated as the cumulative number of species per belt by means of the function *specnumber* in the vegan R package [41]. We used a one-way analysis of variance to test whether the species richness differed between bedrocks. Moreover, a linear model was used to test the elevation–species richness relationship. All the analyses were performed in R 4.3.2 [42].

For each bedrock, we calculated the total beta diversity (βtot), as well as its partition in species replacement (βrepl) and richness difference (βrich), referring to the POD framework [43] and using the BAT package [44] and the Sørensen index. We then tested the relationship between the three components of beta diversity and the elevational gradient through Mantel tests, using Pearson’s correlation coefficient. The Euclidean distance between the elevational belts was used as the environmental distance. We used the *mantel* function in the vegan package [41] with 9999 permutations to estimate the statistical significance of the correlation [45].

To better elucidate the mechanisms underlying lichen diversity patterns, we considered both the affinity of the species to temperature and functional traits related to climate. Lichen species were assigned to two categories on the basis of their ecological affinity to temperature [22]: (a) cryophilous, including cold-adapted and strictly arctic-alpine species (e.g., *Dacampia hookeri* (Borrer) A. Massal., *Nephromopsis nivalis* (L.) Divakar, A. Crespo and Lumbsch, *Stereocaulon alpinum* Laurer); and (b) species with a wide thermal tolerance, including species found in a wider range of temperature conditions (e.g., *Cladonia pyxidata* (L.) Hoffm., *Lecidella elaeochroma* (Ach.) M. Choisy var. *elaeochroma* f. *elaeochroma*, *Psora decipiens* (Hedw.) Hoffm. The classification of lichen species to the different temperature affinity groups was based on the ecological and distributional information available in [46]. For each species, we combined the elevational index, expressed according to an ordinal scale of six values, along with the frequency of observation in different ecoregions of Italy. Species that displayed a broad altitudinal range and higher presence in ecoregions associated with warmer climates were assigned to species with wide thermal tolerance. Conversely, lichens mainly occurring at higher elevations in alpine and subalpine ecoregions were assigned to cryophilous. We used a one-way analysis of variance to test whether the proportion of cryophylous species differed between bedrocks. In addition, a linear model was used to test the elevation–cryophylous proportion relationship.

Finally, we used linear regression to test the effects of elevation on the functional diversity of lichen communities. We selected 3 functional traits known to be significantly influenced by environmental gradients in lichens [22,24,47,48], i.e., growth forms, type of photobiont, and reproduction type. We referred to ITALIC [46] to retrieve the functional traits of each species. Subsequently, we used the gowdis function in the FD package [49] to calculate a matrix of functional distances. We then used this matrix, as well as the species-by-site matrix, in the *melodic* function provided by [50] to calculate the mean species pairwise dissimilarity (MPD) based on the occurrence of the species in each elevational belt. We focused on MPD, as it is not correlated with species richness [50]. Finally, we used a one-way analysis of variance to test whether MPD differed between bedrocks. Linear regression was used to test the effects of the interaction of bedrock type and the elevation on the functional diversity. The full list of the recorded species, with both the corresponding categorization into the temperature affinity groups and the functional traits, is provided in Supplementary Materials.

## 3. Results

**Figure 2:**
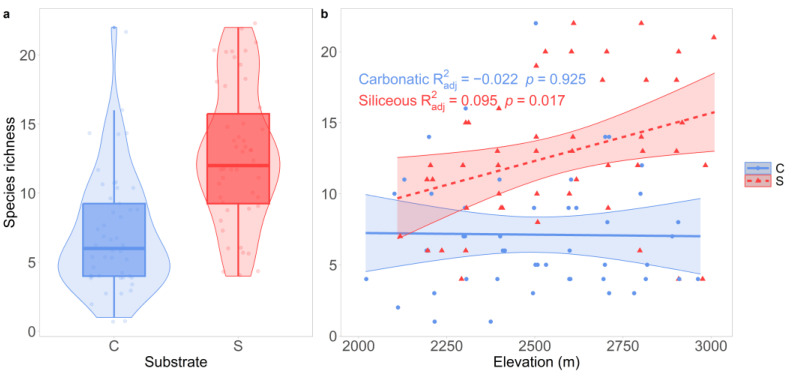
(**a**) Species richness of lichens on carbonatic and siliceous bedrock. (**b**) The relationship between lichen species richness and elevation [m]. The lines show the predicted species richness values, with 95% confidence intervals (bands) according to the linear models. Points represent the observed data. Points represent the observed data. Round points and the solid line represent carbonatic bedrock, while triangles and the dashed line represent siliceous bedrock.

Along the 12 elevational transects, we recorded 126 terricolous lichen species corresponding to approximately 43% of the terricolous lichen biota of the alpine ecoregion of Italy [51]. At the belt level, alpha diversity ranged from 1 to 22 species. In total, 63 species were found on carbonatic and 91 on siliceous bedrock. Twenty-nine species were shared between bedrocks. Species richness was significantly lower (*p* < 0.001) on carbonatic (7.12 ± 4.22, Figure 2a) than on siliceous (12.8 ± 4.98, Figure 2a) bedrock. The species–elevation relationship was non-significant (*p* = 0.925) on carbonatic bedrock, while it was significantly positive on siliceous bedrock (*p* = 0.017, Figure 2b).

**Figure 3:**
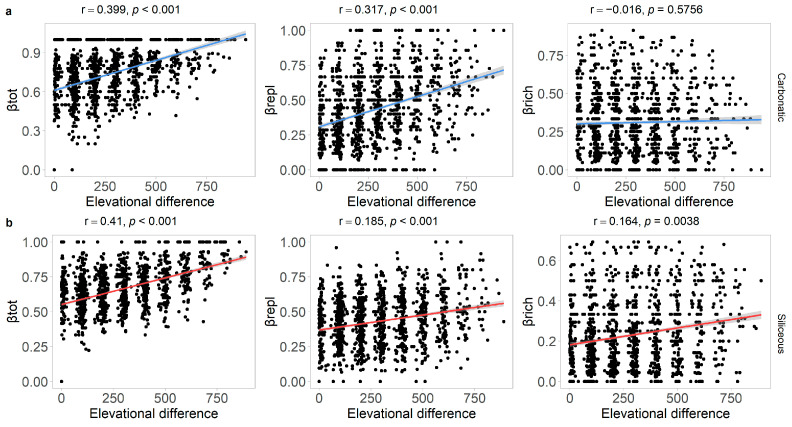
Relationship between the three facets of beta diversity and elevational difference [m] on carbonatic ((**a**) blue regression line) and siliceous ((**b**) red regression line) bedrock. The three facets of beta diversity were total beta diversity (βtot), its partition in species replacement (βrepl), and richness difference (βrich).

The results of the Mantel test showed that, on both carbonatic and siliceous bedrock, βtot was positively related to the elevational difference (Table 1). Similarly, βrepl was positively related to the elevational difference on both bedrocks, with a stronger relationship on carbonatic than on siliceous bedrock. In contrast, βrich was significantly correlated with the elevational difference only on siliceous bedrock (Table 1, Figure 3).

**Figure 4:**
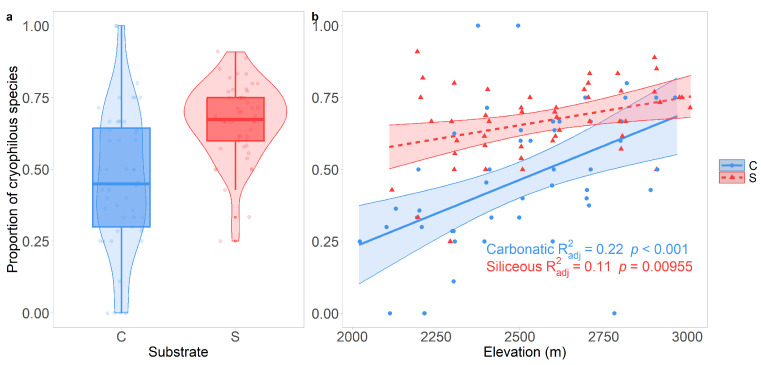
(**a**) Proportion of cryophilous lichen species on carbonatic and siliceous bedrock. Functional traits investigated were growth form, photobiont type, and type of reproduction. Functional diversity was quantified as the mean species pairwise dissimilarity (MPD) based on the occurrence of the species in each elevational belt. (**b**) Relationship between the proportion of cryophilous species and elevation [m]. Lines show the predicted proportion of cryophilous species values with 95% confidence intervals (bands) according to the linear models. Points represent the observed data. Round points and the solid line represent carbonatic substrata, while triangles and the dashed line represent siliceous substrata.

The proportion of cryophilous species was significantly lower (*p* = 0.018) on carbonatic (0.47 ± 0.24, Figure 4a) than on siliceous bedrock (0.67 ± 0.13, Figure 4a), and it was positively related (*p* < 0.001 on carbonatic and *p* = 0.0096 on siliceous bedrock) to elevation on both types of bedrock (Figure 4b), albeit with a steeper increment on carbonatic bedrock.

**Figure 5:**
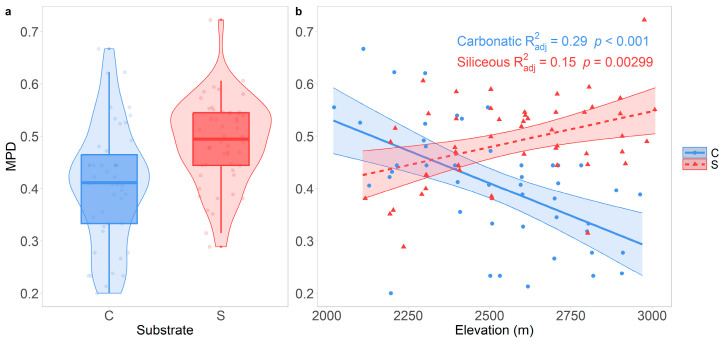
(**a**) Functional diversity on carbonatic and siliceous bedrock. (**b**) The relationship between functional diversity and elevation [m]. Lines show the predicted functional diversity values with 95% confidence intervals (bands) according to the linear models. Points represent the observed data. Round points and the solid line represent carbonatic bedrock, while triangles and the dashed line represent siliceous bedrock.

Finally, MPD was significantly lower (*p* < 0.001) on carbonatic (0.408 ± 0.11, Figure 5a) than on siliceous bedrock (0.489 ± 0.082, Figure 5a). The MPD–elevation relationship was significantly negative (*p* < 0.001) on carbonatic bedrock and positive (*p* = 0.0029) on siliceous bedrock (Figure 5b).

## 4. Discussion

To the best of our knowledge, this is the first study that has quantitatively compared terricolous lichen diversity patterns between carbonatic and siliceous bedrock in the Alps from a climate change perspective. While the study was not specifically devoted to maximizing species capture, our sampling design allowed us to detect a relatively high proportion of the terricolous lichen biota of the Alpine ecoregion of Italy [51], thus reinforcing the generalization of our results, which clearly reveal a bedrock-dependent effect of climate change on the terricolous lichen communities of the Alps. As hypothesized, siliceous bedrocks host a richer lichen biota than carbonatic bedrocks, reflecting a general richness pattern at the national level [52], and seem to be less prone to rapid pauperization of the lichen biota, which can be related to climate change.

On carbonatic bedrock, lichen richness is not affected by elevation, and the lichen biota is likely composed of relatively stenotherm species that change along the elevational gradient according to climatic conditions, resulting in species compartmentalization along the gradient. Actually, compositional differences along the elevational gradient are almost exclusively generated by a species substitution mechanism. This, however, may imply more rapid and irreversible impact of warming, especially on cold-adapted species currently related to high altitudes on carbonatic bedrock, which may face rapid local extinction, likely being replaced by species with wide thermal tolerance. This view is corroborated by the steep cryophylous–elevation relationship, similarly to what has already been recorded for vascular plants [22,53]. In this perspective, cold-adapted species on carbonatic bedrock are expected to persist only in the short term in the higher part of the elevational gradient as a residual component of the lichen biota adapted to cold conditions. Moreover, the steep negative functional diversity–elevation relationship indicates that the functional diversity at high elevation on carbonatic bedrock is extremely low, suggesting that here, species share a few selected traits (i.e., strong ecological filter) that still allow for their persistence under increasingly unfavourable conditions. As in the case of genetic bottlenecks related to reduced population size and genome pauperization that may result in local species extinction [54], this situation could be interpreted as a functional bottleneck with trait pauperization, which could precede the imminent extinction of the cold-adapted lichen biota. Similar results were reported for carbonatic mountains of the Mediterranean area [22,47], indicating a particularly critical general situation for terricolous lichens at high elevation ranges on carbonatic bedrock in a climate change scenario.

In contrast, on siliceous bedrock, species richness increases with elevation, and both species replacement and species gain and loss, determining richness differences, contribute to compositional changes, suggesting that climate warming may simultaneously cause the loss and the substitution of species. However, the cryophylous–elevation relationship for siliceous sites suggests that the local extinction of the more cold-adapted species may be slower than on carbonatic bedrocks, as indicated by a less steep slope of the model, showing that cryophilous species represent a relevant part of the lichen biota even at relatively low elevation. In this scenario, we could hypothesize a longer persistence (e.g., through upward migration of species that currently occur also in the lower part of the elevational gradient) of cold-adapted species that can be mediated by more favourable conditions for terricolous lichens on siliceous bedrock as compared to carbonatic bedrock. In this perspective, siliceous bedrock would likely provide more suitable climatic refugia [55,56,57] that can mitigate the effects of climate warming on terricolous lichens and ensure longer relaxation times before the extinction debt related to changes in macroclimatic conditions would be paid [58]. This pattern is paralleled by that of the functional diversity–elevation relationship, which indicates moderate loss of functional diversity with climate warming. This would imply the maintenance of diverse traits which can ensure greater resilience and resistance to climate-related perturbations. At lower elevations, the communities might persist in the actual state if the tolerance of thermophilous species is wider than cryophilous. On the other hand, the upward shift of some types of vegetation, such as dense herbaceous formations or even forests, may lead to the loss of present communities with the substitution of total different communities, likely dominated by vascular plants.

## 5. Conclusions

In conclusion, this work contributes to elucidating the potential bedrock-mediated response to climate change of terricolous lichens in the Alps, indicating that the most cold-adapted part of this biota could be especially threatened in the next future, mainly on carbonatic bedrock. This pattern could be exacerbated by the increasing human activity at increasingly higher elevations related to summer and winter tourism. Especially for winter tourism, the development of increasingly specialized and high-altitude ski runs and roads [59,60] implies huge soil movements that can inevitably damage this vulnerable biota. In this perspective, carbonatic areas such as the Dolomites, which will host the next Olympic Games in 2026, appear to be particularly sensitive. 

## Figures and Tables

**Table 1:** Mantel test relationships between community composition and elevation. Significant values are marked in bold. The total beta diversity (βtot) is the total variation in composition between communities, considering both species replacement and species loss or gain. The replacement component (βrepl) describes the beta diversity attributed solely to species replacement, while the richness difference component (βrich) reflects the variation due to species loss or gain.

	Carbonatic	Siliceous
βtot	**r = 0.399, *p* = 0.001**	**r = 0.408, *p* = 0.001**
βrepl	**r = 0.317, *p* = 0.001**	**r = 0.185, *p* = 0.004**
βrich	r = −0.01, *p* = 0.981	**r = 0.164, *p* = 0.0026**

## Data Availability

The raw data supporting the conclusions of this article will be made available by the authors upon request.

## Supplementary Materials

The following supporting information can be downloaded at: https://www.mdpi.com/article/10.3390/jof10120836/s1, Table S1. The list of species identified and analysed in the work. We assigned three types of functional traits for each species and two temperature affinity groups. According to Growth Form (Gr.F) species were divided in Squamulous (Sq), Crustuse (Cr), Foliose (Fo) and Fruticose (Fr) forms. According to Photosynthetic Partner (Pho) species were divided respectively in species with only cyanobacteria (Cy) or green algae (Ch) both cyanobacteria and green algae (Ch-Cy.h) as photobiont. According to the reproductive Strategy (Repr) species were divided into mainly sexual (S), mostly asexual, by soredia, or soredia-like structures (A.s), mainly asexual, by isidia, or isidia-like structures (A.i); mainly asexual, by thallus fragmentation (A.f). Temperature affinities divide species in Cryophilous, including cold-adapted and strictly arctic-alpine species and species with wide thermal tolerance (Wide), including species found in a wider range of temperature conditions.
