# Supplementary material for: Bedrock-Dependent Effects of Climate Change on Terricolous Lichens Along Elevational Gradients in the Alps
Source: J Fungi (Basel). 2024 Dec 3;10(12):836. doi: 10.3390/jof10120836 (PMC11678017; doi:10.3390/jof10120836)
Supplement: Supplementary file 1 [file jof-10-00836-s001.zip › jof-3301883-supplementary.pdf]

Supplementary Materials

Table S1. The list of species identified and analysed in the work. We assigned three types of functional traits for each species and two temperature affinity groups. According to Growth Form (Gr.F) species were divided in Squamulous (Sq), Crustuse (Cr), Foliose (Fo) and Fruticose (Fr) forms. According to Photosynthetic Partner (Pho) species were divided respectively in species with only cyanobacteria (Cy) or green algae (Ch) both cyanobacteria and green algae (Ch-Cy.h) as photobiont. According to the reproductive Strategy (Repr) species were divided into mainly sexual (S), mostly asexual, by soredia, or soredia-like structures (A.s), mainly asexual, by isidia, or isidia-like structures (A.i); mainly asexual, by thallus fragmentation (A.f). Temperature affinities divide species in Cryophilous, including cold-adapted and strictly arctic-alpine species and species with wide thermal tolerance (Wide), including species found in a wider range of temperature conditions.

Scientific name	**Gr.F**	**Pho**	**Repr**	**Temp. Aff.**
Agonimia gelatinosa (Ach.) M. Brand & Diederich	Cr	Ch	S	Cryophilous
Agonimia tristicula (Nyl.) Zahlbr.	Sq	Ch	S	Wide
Alectoria nigricans (Ach.) Nyl.	Fr	Ch	A.f	Cryophilous
Alectoria ochroleuca (Hoffm.) A. Massal.	Fr	Ch	A.f	Cryophilous
Arthrorhaphis alpina (Schaer.) R. Sant.	Cr	Ch	S	Cryophilous
Arthrorhaphis citrinella (Ach.) Poelt	Cr	Ch	S	Cryophilous
Athallia saxifragarum (Poelt) Arup, Frödén & Søchting	Cr	Ch	S	Cryophilous
Baeomyces carneus (Retz.) Flörke	Cr	Ch	S	Cryophilous
Baeomyces placophyllus Ach.	Cr	Ch	S	Cryophilous
Biatora subduplex (Nyl.) Printzen	Cr	Ch	S	Cryophilous
Biatora vernalis (L.) Fr.	Cr	Ch	S	Wide
Bilimbia lobulata (Sommerf.) Hafellner & Coppins	Cr	Ch	S	Wide
Bilimbia microcarpa (Th. Fr.) Th. Fr.	Cr	Ch	S	Cryophilous
Bilimbia sabuletorum (Schreb.) Arnold	Cr	Ch	S	Wide
Blastenia ammiospila (Ach.) Arup, Søchting & Frödén	Cr	Ch	S	Cryophilous
Brodoa intestiniformis (Vill.) Goward	Fo	Ch	S	Cryophilous
Bryobilimbia hypnorum (Lib.) Fryday, Printzen & S. Ekman	Cr	Ch	S	Wide
Bryonora castanea (Hepp) Poelt	Cr	Ch	S	Cryophilous
Bryoplaca sinapisperma (DC.) Søchting, Frödén & Arup	Cr	Ch	S	Cryophilous
Caloplaca stillicidiorum (Vahl) Lynge	Cr	Ch	S	Cryophilous
Catapyrenium cinereum (Pers.) Körb.	Sq	Ch	S	Cryophilous
Cetraria commixta (Nyl.) Th. Fr.	Fo	Ch	S	Cryophilous
Cetraria ericetorum Opiz	Fr	Ch	A.f	Cryophilous
Cetraria islandica (L.) Ach. subsp. islandica	Fr	Ch	A.f	Wide

Cetraria juniperina (L.) Ach.	Fo	Ch	A.f	Cryophilous
Cetraria muricata (Ach.) Eckfeldt	Fr	Ch	S	Wide
Cladonia arbuscula (Wallr.) Flot.	Fr	Ch	A.f	Cryophilous
Cladonia borealis S. Stenroos	Sq	Ch	A.s	Cryophilous
Cladonia cariosa (Ach.) Spreng.	Sq	Ch	S	Cryophilous
Cladonia carneola (Fr.) Fr.	Sq	Ch	A.s	Cryophilous
Cladonia chlorophaea (Sommerf.) Spreng.	Sq	Ch	A.s	Wide
Cladonia coccifera (L.) Willd.	Sq	Ch	A.s	Cryophilous
Cladonia deformis (L.) Hoffm.	Sq	Ch	A.s	Cryophilous
Cladonia ecmocyna Leight.	Sq	Ch	S	Cryophilous
Cladonia fimbriata (L.) Fr.	Sq	Ch	A.s	Wide
Cladonia firma (Nyl.) Nyl.	Sq	Ch	S	Wide
Cladonia foliacea (Huds.) Willd. f. foliacea	Fr	Ch	S	Wide
Cladonia furcata (Huds.) Schrad. subsp. furcata	Fr	Ch	S	Wide
Cladonia gracilis (L.) Willd.	Sq	Ch	S	Cryophilous
Cladonia macroceras (Delise) Hav.	Sq	Ch	S	Cryophilous
Cladonia macrophyllodes Nyl.	Sq	Ch	S	Cryophilous
Cladonia mitis Sandst.	Fr	Ch	A.f	Cryophilous
Cladonia pleurota (Flörke) Schaer.	Sq	Ch	A.s	Cryophilous
Cladonia pyxidata (L.) Hoffm.	Sq	Ch	A.f	Wide
Cladonia pyxidata (L.) Hoffm. f. pocillum (Ach.) Nyl.	Sq	Ch	S	Wide
Cladonia rangiferina (L.) F.H. Wigg.	Fr	Ch	A.f	Cryophilous
Cladonia squamosa Hoffm. var. squamosa	Fr	Ch	A.i	Wide
Cladonia stellaris (Opiz) Pouzar & Vězda	Fr	Ch	S	Cryophilous
Cladonia symphycarpa (Flörke) Fr.	Sq	Ch	S	Wide
Cladonia uncialis (L.) F.H. Wigg. subsp. uncialis	Fr	Ch	S	Cryophilous
Cladonia verticillata (Hoffm.) Schaer.	Fr	Ch	S	Wide
Dacampia hookeri (Borrer) A. Massal.	Cr	Ch	S	Cryophilous
Dactylina ramulosa (Hook. f.) Tuck.	Fr	Ch	A.f	Cryophilous
Dibaeis baeomyces (L. f.) Rambold & Hertel	Cr	Ch	S	Cryophilous
Enchylium tenax (Sw.) Gray	Fo	Cy	S	Wide
Fuscopannaria praetermissa (Nyl.) P.M. Jørg.	Sq	Cy	A.s	Cryophilous

Helocarpon pulverulum (Th. Fr.) Türk & Hafellner	Cr	Ch	S	Cryophilous
Heppia adglutinata (Kremp.) A. Massal.	Sq	Cy	S	Wide
Lathagrium undulatum (Flot.) Poetsch	Fo	Cy	S	Wide
Lecania subfuscula (Nyl.) S. Ekman	Cr	Ch	S	Wide
Lecanora epibryon (Ach.) Ach. var. epibryon	Cr	Ch	S	Cryophilous
Lecidella elaeochroma (Ach.) M. Choisy var. elaeochroma f. elaeochroma	Cr	Ch	S	Wide
Lecidella wulfenii (Hepp) Körb.	Cr	Ch	S	Cryophilous
Lecidoma demissum (Rutstr.) Gotth. Schneid. & Hertel	Cr	Ch	S	Cryophilous
Lepraria alpina (B. de Lesd.) Tretiach & Baruffo	Lepr	Ch	A.s	Cryophilous
Lepraria borealis Loht. & Tønsberg	Lepr	Ch	A.s	Wide
Lepraria caesioalba (B. de Lesd.) J.R. Laundon	Lepr	Ch	A.s	Wide
Lepraria diffusa (J.R. Laundon) Kukwa	Lepr	Ch	A.s	Wide
Lepraria eburnea J.R. Laundon	Lepr	Ch	A.s	Wide
Lepraria finkii (B. de Lesd.) R.C. Harris	Lepr	Ch	A.s	Wide
Lepraria neglecta (Nyl.) Erichsen	Lepr	Ch	A.s	Cryophilous
Lepraria vouauxii (Hue) R.C. Harris	Lepr	Ch	A.s	Wide
Lobaria linita (Ach.) Rabenh.	Fo	Ch	A.s	Cryophilous
Megaspora verrucosa (Ach.) Arcadia & A. Nordin	Cr	Ch	S	Cryophilous
Mycobilimbia sphaeroides (Dicks.) S. Ekman & Printzen	Cr	Ch	S	Wide
Mycobilimbia tetramera (De Not.) Hafellner & Türk	Cr	Ch	S	Wide
Myriolecis zosterae (Ach.) Śliwa, Zhao Xin & Lumbsch var. palanderi (Vain.) Śliwa	Cr	Ch	S	Cryophilous
Nephroma expallidum (Nyl.) Nyl.	Fo	Ch	S	Cryophilous
Nephroma parile (Ach.) Ach.	Fo	Cy	A.s	Cryophilous
Nephromopsis cucullata (Bellardi) Divakar, A. Crespo & Lumbsch	Fr	Ch	A.f	Cryophilous
Nephromopsis nivalis (L.) Divakar, A. Crespo & Lumbsch	Fr	Ch	A.f	Cryophilous
Parmelia sulcata Taylor	Fo	Ch	A.s	Wide
Parvoplaca tiroliensis (Zahlbr.) Arup, Søchting & Frödén	Cr	Ch	S	Cryophilous
Peltigera aphthosa (L.) Willd.	Fo	Ch-Cy.h	S	Cryophilous
Peltigera didactyla (With.) J.R. Laundon	Fo	Cy	A.s	Wide
Peltigera extenuata (Vain.) Lojka	Fo	Cy	A.s	Wide
Peltigera lepidophora (Vain.) Bitter	Fo	Cy	A.i	Cryophilous

Peltigera leucophlebia (Nyl.) Gyeln.	Fo	Ch-Cy.h	S	Cryophilous
Peltigera malacea (Ach.) Funck	Fo	Ch	S	Cryophilous
Peltigera ponojensis Gyeln.	Fo	Cy	S	Cryophilous
Peltigera praetextata (Sommerf.) Zopf	Fo	Cy	A.i	Wide
Peltigera rufescens (Weiss) Humb.	Fo	Cy	S	Wide
Peltigera venosa (L.) Hoffm.	Fo	Ch	S	Cryophilous
Pertusaria geminipara (Th. Fr.) Brodo	Cr	Ch	A.s	Cryophilous
Pertusaria oculata (Dicks.) Th. Fr.	Cr	Ch	A.i	Cryophilous
Physconia muscigena (Ach.) Poelt var. muscigena	Fo	Ch	S	Cryophilous
Placidium squamulosum (Ach.) Breuss	Sq	Ch	S	Wide
Placynthiella icmalea (Ach.) Coppins & P. James	Cr	Ch	A.i	Wide
Polyblastia sendtneri Kremp.	Cr	Ch	S	Cryophilous
Protopannaria pezizoides (Weber) P.M. Jørg. & S. Ekman	Cr	Cy	S	Cryophilous
Protothelenella sphinctrinoides (Nyl.) H. Mayrhofer & Poelt	Cr	Ch	S	Cryophilous
Pseudephebe pubescens (L.) M. Choisy	Fr	Ch	A.f	Cryophilous
Psora decipiens (Hedw.) Hoffm.	Sq	Ch	S	Wide
Psoroma tenue Henssen var. boreale Henssen	Cr	Ch	S	Cryophilous
Pycnothelia papillaria Dufour	Fr	Ch	S	Wide
Rinodina conradii Körb.	Cr	Ch	S	Wide
Rinodina mniaraea (Ach.) Körb.	Cr	Ch	S	Cryophilous
Rinodina olivaceobrunnea C.W. Dodge & G.E. Baker	Cr	Ch	S	Cryophilous
Rinodina roscida (Sommerf.) Arnold	Cr	Ch	S	Cryophilous
Scutula igniarii (Nyl.) S. Ekman	Cr	Ch	S	Wide
Scytinium imbricatum (P.M. Jørg.) Otálora, P.M. Jørg. & Wedin	Sq	Cy	S	Cryophilous
Solorina bispora Nyl. subsp. bispora	Fo	Ch	S	Cryophilous
Solorina crocea (L.) Ach.	Fo	Ch	S	Cryophilous
Solorina octospora (Arnold) Arnold	Fo	Ch	S	Cryophilous
Stereocaulon alpinum Laurer	Fr	Ch-Cy.h	S	Cryophilous
Tetramelas geophilus (Sommerf.) Norman	Cr	Ch	S	Cryophilous
Tetramelas insignis (Hepp) Kalb	Cr	Ch	S	Cryophilous
Tetramelas thiopolizus (Nyl.) Giralt & P. Clerc	Cr	Ch	S	Cryophilous
Thamnolia vermicularis (Sw.) Schaer. var. vermicularis	Fr	Ch	A.f	Cryophilous

Thelidium zwackhii (Hepp) A. Massal.	Cr	Ch	S	Wide
Toniniopsis bagliettoana (A. Massal. & De Not.) Kistenich & Timdal	Cr	Ch	S	Wide
Trapeliopsis gelatinosa (Flörke) Coppins & P. James	Cr	Ch	S	Cryophilous
Trapeliopsis granulosa (Hoffm.) Lumbsch	Cr	Ch	S	Cryophilous
Umbilicaria cylindrica (L.) Delise var. cylindrica	Fo	Ch	S	Cryophilous
Umbilicaria decussata (Vill.) Zahlbr.	Fo	Ch	A.s	Cryophilous
Xylographa parallela (Ach.) Fr.	Cr	Ch	S	Wide
